# Tuft cell integration of luminal states and interaction modules in tissues

**DOI:** 10.1007/s00424-021-02630-2

**Published:** 2021-10-11

**Authors:** Christoph Schneider

**Affiliations:** Institute of Physiology, University of Zurich, Winterthurerstrasse 190, CH-8057 Zürich, Switzerland

**Keywords:** Tuft cells, Aversive responses, Type 2 immunity, Chemosensation

## Abstract

Chemosensory processes are integral to the physiology of most organisms. This function is typically performed by specialized cells that are able to detect input signals and to convert them to an output dedicated to a particular group of target cells. Tuft cells are cholinergic chemosensory epithelial cells capable of producing immunologically relevant effector molecules. They are scattered throughout endoderm-derived hollow organs and function as sensors of luminal stimuli, which has been best studied in mucosal barrier epithelia. Given their epithelial origin and broad distribution, and based on their interplay with immune pathways, tuft cells can be considered a prototypical example of how complex multicellular organisms engage innate immune mechanisms to modulate and optimize organ physiology. In this review, I provide a concise overview of tuft cells and discuss how these cells influence organ adaptation to dynamic luminal conditions.

## Introduction

Most organs in the body rely on chemosensory processes in order to respond appropriately to their environment. This is often enabled by specialized chemosensory cells that are capable of both detecting environmental cues and converting these inputs into usable signals that surrounding non-chemosensory cells can respond to. Over the past several decades, research by physiologists has identified tuft cells as important players in the chemosensation signal transduction pathway. Tuft cells, which are also known as brush cells, microvillous cells, caveolated cells, or solitary chemosensory cells, are relatively rare cholinergic chemosensory epithelial cells that are found in multiple different organs and that play a role in signal transduction across barrier epithelia. Tuft cells have been found to engage in innate immune responses to various microbial stimuli, a finding that has captured the attention of immunologists. Despite the recent interest in these fascinating epithelial chemosensory cells, the biology of tuft cells still remains relatively enigmatic. In this review, I provide an overview of tuft cell biology and summarize evidence for tuft cells contributing to both aversive reflexes and barrier tissue adaptation in response to changing environmental conditions.

Historically, tuft cells were identified in epithelia of hollow organs by their apical microvillus-rich tuft with dense actin filaments and deep-reaching rootlets. These structures can be visualized by phalloidin, and morphologically distinguish tuft cells from other surrounding epithelial cells [1, 2]. In addition, tuft cells are enriched for a number of markers that are structural proteins or associated with structural proteins. Such markers include villin [3], advillin [4], and β-tubulin [5], which are all enriched in the apical tuft, as well as cytokeratin-18 [6] and the microtubule-associated serine/threonine-protein kinase DCLK1 (doublecortin-like and CAM kinase-like 1) [7]. A seminal finding by Höfer, Püschel, and Drenckhahn demonstrated that tuft cells in the intestine also express α-gustducin (guanine nucleotide-binding protein G(t) subunit α3; GNAT3) [8]. GNAT3 is a protein critically involved in the canonical taste transduction cascade employed by type II taste cells in the oral cavity, which detect sweet, bitter, and umami tastants. This surprising finding sparked subsequent studies by many groups, revealing that tuft cells across various organs express signaling components of the canonical taste transduction cascade (reviewed in [9]). In addition to GNAT3, these tuft cell-expressed signaling components include phospholipase Cβ2 (PLCβ2), the ER membrane calcium channel inositol-1,4,5-trisphosphate receptor (InsP3R), and the depolarizing calcium-activated cation channel TRPM5. Recent studies found that this expression program is relatively conserved in humans, including in tuft cells from the intestine [10–12].

Despite being able to identify tuft cells by both morphology and key protein markers, the developmental pathway associated with the tuft cell lineage is not well characterized. Aside from tuft cells’ strict reliance on the lineage-defining transcription factor POU2F3—another characteristic shared with type II taste cells—very little is known about the process of tuft cell lineage commitment. Interestingly, a number of additional transcription factors were found to be enriched in tuft cells relative to other epithelial cells, including Gfi-1b, SOX-9, and SPI-B. Their function, however, remains unclear. It is possible that these transcription factors are part of the machinery that regulates the differentiation from epithelial progenitors and the subsequent expression of the core tuft cell transcriptional program, a program which is overall strongly conserved in most tissues [13]. The striking similarities in gene expression among different organ-specific tuft cells suggest a shared core function. All tuft cells seem capable of detecting luminal (microbial) stimuli and relaying signals via taste signaling components, resulting in the release of appropriate effector molecules to not only neighboring epithelial cells but also to subepithelial immune and non-immune cells. While the core signaling pathways are highly conserved on a transcriptional level [13], there appears to be more diversity in the nature of the upstream stimuli that are detected, as well as the downstream effector modules. A brief summary of currently known physiological ligand-receptor pairs for tuft cells in different organs is provided in Table 1. It should be noted, however, that one has to be careful with this type of generalized conclusion regarding tuft cell biology, especially since most studies focused only on one particular organ.

**Table 1:** Ligand-receptor pairs for tuft cells in different organs. Other ligands with no physiologically known source have been used to stimulate tuft cells in various organs and include bitter taste receptor agonists, most commonly denatonium [14, 15]. *, known receptors and inferred by expression data but lacking evidence from loss of function experiments

Ligand (source)	Receptor	Organ	Physiological consequences	References
*N*-formyl methionine- containing peptides (bacteria, predominantly *Enterobacteriaceae*)	Unknown receptor (formyl peptide receptor-independent)	Trachea	Enhanced mucociliary clearance	[16]
2-Heptyl-3-hydroxy-4-quinolone (*P. aeruginosa* quinolone signal)	Tas2Rs*	Trachea	Enhanced mucociliary clearance	[17]
3-oxo-C12-homoserine lactone (HSL), 3-oxo-C6-HSL (bacterial quorum sensing molecules, including from *P. aeruginosa*, *E. coli*)	Tas2Rs*	Nasal respiratory epithelium, trachea	Trigeminally mediated apnea, enhanced mucociliary clearance	[18–20]
ATP (unknown source; likely endogenous)	Purinergic receptor P2Y2	Nasal epithelium	Cysteinyl leukotriene release in tuft cells; ATP also acts on non-tuft cells	[15, 21, 22]
Succinate (*Tritrichomonas* protists; bacteria)	Succinate receptor 1	Small intestine	IL-25-dependent tuft cell–ILC2 circuit activation	[13, 23–25]

**Fig. 1:**
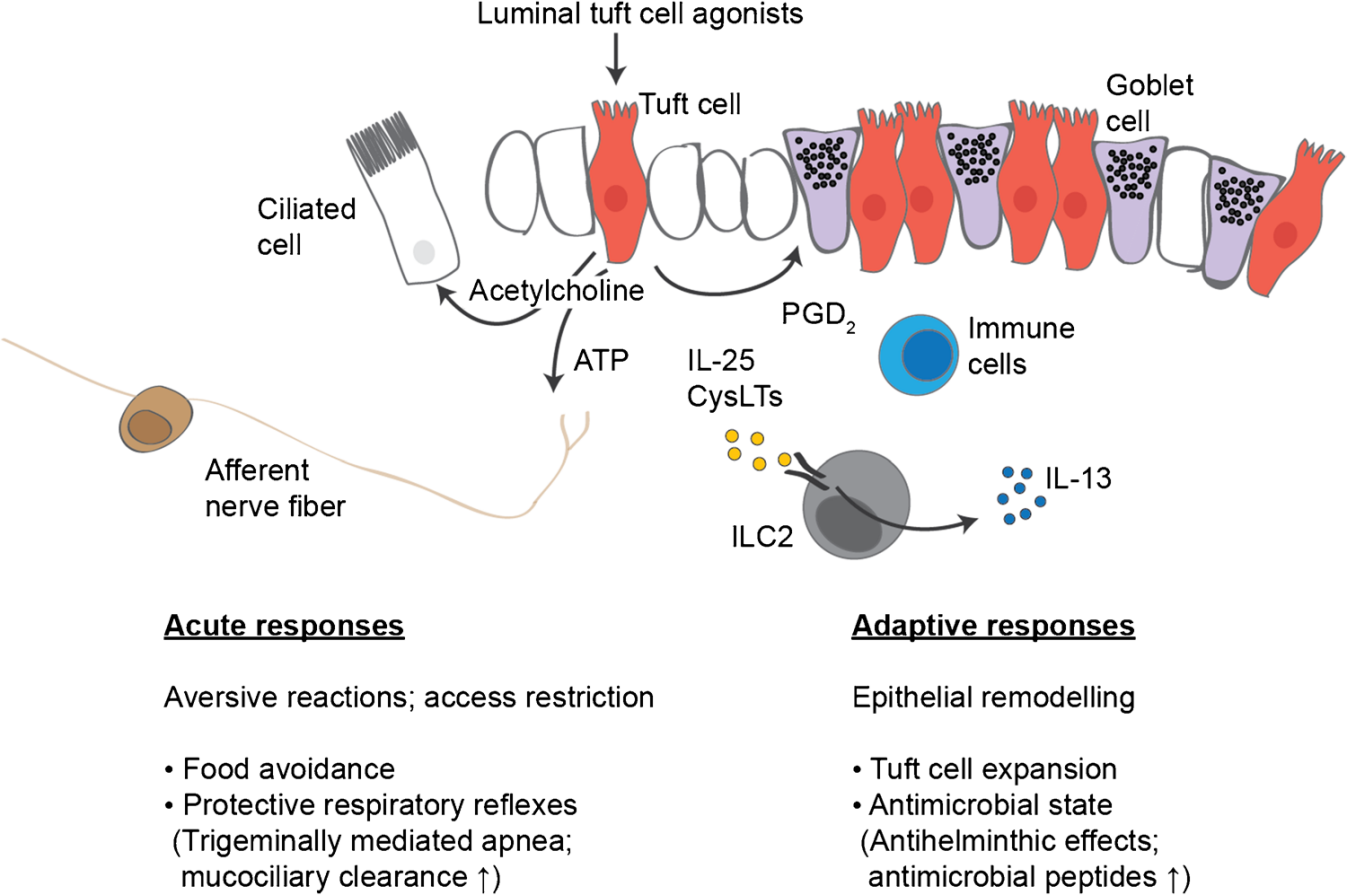
Summary of tuft cell interaction modules. Stimulation of tuft cells with luminal agonists triggers the release of one or multiple tuft cell effector molecules in a process that remains incompletely characterized. Known effector molecules include acetylcholine (various tissues), ATP (type II taste cells), IL-25 (small intestine), cysteinyl leukotrienes (CysLTs) (small intestine, airways), and prostaglandin D_2_ (PGD_2_) (small intestine, pancreas). Acetylcholine has a relatively broad action profile and is primarily associated with acute aversive responses in multiple organs via effects on ciliated cells and afferent nerve fibers (see text). ATP functions as a critical second messenger for the signaling of gustatory information from type II taste cells to afferent fibers in the oral taste buds [30]. IL-25 and CysLTs cooperatively stimulate the production of IL-13 and other type 2 cytokines in group 2 innate lymphoid cells (ILC2s) of the small intestine lamina propria (see text). Recent studies revealed roles of PGD_2_ in injury-associated pancreatic tumorigenesis [31] and small intestinal type 2 responses [32] through effects on incompletely defined immune and epithelial cell populations. IL-25 and tuft cell-derived eicosanoids appear to be predominantly involved in the regulation of epithelial remodeling

A number of recent comprehensive reviews illuminated diverse aspects of tuft cell biology [26–29]. In the following sections, I will focus on currently known roles of tuft cells in aversive and adaptive responses in tissues. A summary of some of these interaction modules is provided in Fig. 1.

## Tuft cells in acute aversive responses

The responses that tuft cells elicit in their interaction partners are suggested to be qualitatively analogous to those of taste cells stimulated with bitter tastants. This is supported by similarities in pathways engaged by tuft cells and type II taste cells, and the expression of certain bitter taste receptors (Tas2Rs) by some tuft cells. The physiological response to substances with a strong bitter taste is rejection, and such aversive behavior is thought to have evolved to protect the organism from potentially toxic molecules [33]. Taste perception, especially detection of a broad range of structurally diverse bitter substances through a large set of Tas2Rs, might be particularly important for omnivorous species. Omnivores diversified their food sources, and in doing so, were faced with both an increased risk of intoxication and a need to identify high caloric (sweet) food [34]. Advanced taste perception and an aversion to bitter tastants provided omnivores with an exquisite hazard-warning system. It would thus not surprise that the same physiology may have been adapted to other organs in an effort to induce similar physiological consequences in response to adverse stimuli. Tuft cells may possibly be an example of this adaptation, as there are many reports of stimulus-provoking agents being rejected based on signaling mediated by tuft cells.

Tuft cell chemosensation appears to be important in protecting the respiratory system. Tuft cells in the nasal epithelium responded to bacterial acyl-homoserine lactones by activating trigeminal afferents to evoke apnea and induce substance P release via cholinergic signaling, which also promoted mast cell degranulation and plasma leakage [14, 19, 20]. Certain bitter substances may be directly sensed by Tas2Rs expressed in ciliated cells of the human upper airways, as exposure to bitter substances resulted in nitric oxide-regulated increases in ciliary beat frequency [35]. In the lower airways, however, such regulation seems to occur on the level of tuft cells, as suggested by two recent studies in mice. Stimulation of tracheal tuft cells with *Pseudomonas* quinolones or formylated peptides engaged signaling via PLCβ2, InsP3R3, and TRPM5, and caused tuft cells to release acetylcholine, which promoted increased ciliary movement in surrounding ciliated cells [16, 17]. These studies reveal mechanistic insight into the involved signaling pathways and expand on prior reports, which demonstrated aversive respiratory responses following stimulation of mouse trachea epithelium with Tas2R agonists; specifically, the respiratory rate dropped and there was increased mucosal surface particle transport speed [18, 36]. Somewhat related, tuft cells at the entrance duct of the mouse vomeronasal organ regulated fluid access into this pheromone-sensing organ in order to protect the organ from harmful or irritating substances [37]. Interestingly, a tuft cell “hot-spot” can also be found in the stomach at the boundary between fundus and corpus, the so-called limiting ridge [38]. Multiple studies reported a decrease in gastric emptying following intragastric administration of bitter receptor agonists, and one study found this effect even more pronounced in *Gnat3*^–/–^ mice [33, 39]. Further studies will help to identify the precise role of gastric tuft cells in regulating access or passage of luminal constituents to the corpus and further to the small intestine. Similarly, stimulation of urethral tuft cells with bitter and umami taste receptor ligands caused acetylcholine release, stimulation of sensory nerve fibers, and increased activity of the bladder detrusor muscle, which may be a protective reflex to flush out microbes and prevent ascending infections [40].

The gastrointestinal tract, in particular the small intestine, is another key location for tuft cell signaling. Several recent studies with a focus on tuft cells in the small intestine identified novel interaction modules with immune cells following detection of luminal parasites by tuft cells. Most notably, a feedforward circuit involving lamina propria-resident group 2 innate lymphoid cells (ILC2s) was shown to be enabled by tuft cell effector molecules such as IL-25 and leukotrienes [29]. Similar to their airway counterparts, small intestinal tuft cells required the canonical taste transduction components, GNAT3, PLCβ2, and TRPM5, for the response to the *Tritrichomonas* protist metabolite succinate downstream of the G protein-coupled receptor SUCNR1 [13, 23–25, 41]. However, SUCNR1 and GNAT3 were dispensable for the sensing of luminal worms which produce ligands and engage receptors of unknown nature [13]. While the tuft cell effector responses are critically involved in the expulsion of helminth model organisms from their intestinal niche, they differ considerably in kinetics when compared to the fast-acting protective and aversive reflexes that are mediated by tuft cells in the other tissues described above. In the *Nippostrongylus brasiliensis* helminth infection model, the peak of the tuft cell–ILC2 response and associated worm clearance is reached approximately 1 week after first exposure to the larvae in the small intestine [42, 43]. As in other tissues, tuft cell activation occurs instantly and secreted effector molecules can be detected a few minutes after stimulation in vitro [44, 45]. Release of preformed mediators from storage compartments could be involved, although evidence for this is currently lacking. It is more likely that some effector molecules may be generated on demand: tuft cells express an enzymatic machinery which enables the rapid synthesis of leukotrienes and other eicosanoid derivatives of arachidonic acid from membrane phospholipids following appropriate stimulation of competent cells [45]. This rapid synthesis is known to be regulated on multiple levels [46].

Il-25 is a key effector molecule for tuft cell function in response to helminth infection. IL-25 transcript, however, is expressed constitutively in tuft cells and remains unchanged upon helminth exposure [43, 45]. Tuft cell stimulation thus might control protein production on the posttranscriptional level, a process known to be used in the regulation of other cytokines [47]. Alternatively, secretion of stored IL-25 protein could be triggered upon tuft cell activation. IL-25 has a secretory signal peptide suggesting a conventional mode of secretion; an inhibitor of this pathway, Brefeldin A, was reported to block activation-induced IL-25 release [44]. Whether IL-25 is rerouted to storage vesicles or directly secreted following translation requires further study. Cleavage of IL-25 by matrix metalloproteinase 7 was reported to further enhance IL-25 activity in asthmatic airways [48]. Additional studies are needed to assess if proteolytic activation of IL-25 is a general mechanism of IL-25 regulation, specifically in the small intestine where functions of IL-25 are well understood.

Overall, tuft cells in the small intestine are capable of responding rapidly to stimuli, but the overall physiological response they invoke is delayed. The interaction module is rather slow between tuft cells that are constantly moving upwards within the epithelial “conveyor belt” along the intestinal crypt-villus axis, and the static ILC2s in the lamina propria. Soluble effectors from tuft cells have to first reach ILC2s, presumably through diffusion. ILC2s then have to induce their own transcriptional effector program that includes the canonical type 2 cytokines IL-5, IL-9, and IL-13, which are transcribed from readily accessible loci [49, 50]. Although studies using transcriptional reporter alleles have demonstrated that *Il13* expression can be detected in ILC2s less than 24 h after oral gavage of helminths [45, 51], mounting a potent type 2 immune response appears to require continuous cross-talk between tuft cells, ILC2s, and the crypt epithelial progenitor compartment to alter epithelial composition (discussed in more detail below). While additional and more acute responses are conceivable, including communication with adjacent epithelial cells, stimulation of enteric neurons, and hypercontractility of smooth muscle, further study is warranted to establish a direct role of tuft cells in these processes.

When considering the tuft cell modules and defensive/aversive strategies of the gastrointestinal tract, interesting parallels with food allergy reactions emerge. Innocuous dietary antigens can elicit acute responses that have a strong type 2 immune component and involve players such as immunoglobulin E (IgE), mast cells, and basophils [52]. Physiological responses to these dietary antigens in the intestine include elevated mucus secretion, smooth muscle contractions, and acute diarrhea. It has been speculated that these are coordinated by a gastrointestinal food quality control system that can be trained to identify negative food value by associating adverse consequences of a harmful substance with a particular dietary constituent (antigen sensitization) [53]. Analogous to conditioned taste aversion (CTA), in which adverse consequences are associated with gustatory or olfactory cues in food [33], intestinal antigen sensitization (conditioning) and detection with high specificity upon subsequent re-exposure enables appropriate responses geared towards ridding the intestine of noxious chemicals. Such an alternative defensive strategy might provide another layer of protection and particularly benefit the organism when CTA is insufficient, perhaps due to absence of a strong taste or flavor or when such signals are outweighed in food with a very positive valence (e.g., a combination of high caloric food and an energy-deprived metabolic state). In exaggerated form and perhaps with hypersensitive predispositions, these physiological defense strategies can manifest as a food allergy [53]. More generally, it has been hypothesized that mechanisms of allergic (type 2 immune) responses have evolved as part of a complex physiological system that is particularly active at barrier sites and dedicated to aversion and expulsion of noxious substances, specifically environmental toxins [54]. This defensive system can cause undesired and pathological symptoms when unbalanced or targeted at a substance associated with common environmental antigens.

It will be interesting if and how tuft cell chemosensation and effector molecules are engaged in such responses at the intersection of gustatory and immune sensory mechanisms. A recent study provided some hints that there may be a role for tuft cells in these responses. Using a model of mechanical skin injury, the tuft cell–ILC2 circuit promoted mast cell expansion in the small intestine, which potentiated subsequent anaphylaxis to an oral antigen challenge [55]. Furthermore, data suggesting that activation of skin sensory neurons by leukotrienes can cause acute itch flares offers an opportunity for future studies into the largely unexplored area of intestinal tuft cell-neuron crosstalk via tuft cell-derived eicosanoids [56].

## Tuft cells in tissue adaptation

Acute rejection in response to a stimulus is only one of the possible ways that tuft cells can respond to their environment. Depending on the localization, and perhaps the quality and/or quantity of the stimulus detected by tuft cells, the appropriate response may not be outright rejection of the stimulus, especially when expulsion is not an option. Indeed, an alternative strategy is the tolerization of a stimulus and the adaptation of the tissue to its presence. In humans, for example, there is a cultural history of developing an acquired taste for certain bitter food and drinks. Notably, experimental evidence suggests that a certain level of these “bitter” stimuli is not only tolerated but can have beneficial effects on organism physiology, specifically endocrine homeostasis (summarized in [57]). Integrating such a view into the role of tuft cells in barrier epithelia suggests that a possible favorable consequence of their stimulation with bitter ligands or other tuft cell agonists might be increased resilience to subsequent challenges with the same or related stimulus. This concept is supported by two aspects of tissue alterations often associated with tuft cell activation: an elevated anti-microbial state, and an expanding sensory (i.e., tuft cell) compartment. In most cases, however, the mechanisms that underlie these alterations are not well defined.

### Tuft cells regulating an antimicrobial tissue state

Studies in multiple organs indicate that activated tuft cells are often associated with an elevated antimicrobial state of the local tissue. Stimulating bitter receptors on human sinonasal tuft cells can result in propagation of a calcium-dependent signal to surrounding epithelial cells, causing the release of broad-spectrum antimicrobial substances such as β-defensins 1 and 2 from adjacent epithelial cells [58]. Notably, such a mechanism was not found in cultures from human bronchial epithelium nor mouse nasal epithelium, which suggests possible regional and species-specific tuft cell signaling pathways. Tuft cells in mouse gingival junctional epithelium express several bitter Tas2Rs and their activation results in upregulated expression of β-defensin-3 in gingival tissue, which is abrogated in *Gnat3*^–/–^ mice [59]. Physiological consequences of defective antimicrobial responses in these *Gnat3*^–/–^ mice were found to be an altered oral microbiota and an accelerated alveolar bone loss under homeostatic conditions; pathologies were further exacerbated in a model of ligature-induced periodontitis. For both the gingival tissue and the alveolar bone, the relevant tuft cell effector molecule as well as its precise target cell population are unclear. Because gingival tuft cells lack detectable expression of choline acetyltransferase (ChAT), which can be found in tuft cells of most tissues, they might employ factors other than acetylcholine for communication with their neighboring cells [59].

In the small intestine, Paneth cells constitute an important constitutive source of antimicrobial peptides. Mice deficient in Paneth cell lysozyme display altered gut bacterial landscape, which is associated with increased tuft cell frequencies [60]. There is currently no data, however, that would suggest a direct cross-talk between tuft cells and Paneth cells. The prototypical immune regulators of intestinal antimicrobial proteins are IL-22 and IL-17, although a potential link between the bactericidal protein angiogenin 4 and IL-13 was reported for the colon [61]. Consequences of tuft cells activation in the small intestine are primarily associated with canonical antihelminthic responses [41–43]. These effects are thought to be mediated predominantly by promoting expression of type 2 cytokines in ILC2s, in particular IL-13, which drives goblet cell hyperplasia and the production of mucins and resistin-like molecule-β (RELMβ) [62]. An elevated type 2 immune tone in the small intestine was associated with increased resistance to helminth infection [25], though the individual contribution of different effector mechanisms were not definitively clarified. Similarly, pre-conditioning the intestine by treating mice with effector cytokines IL-25 and IL-4 before or at the time of helminth arrival in the small intestine lumen results in rapid worm clearance [63, 64]. Whether small intestinal tuft cells directly communicate with adjacent epithelial cells and neurons, as their counterparts do in other tissues, requires further study. Notably, a recent study reported that small intestinal tuft cells can directly act on epithelial progenitor cells and goblet cells through secretion of prostaglandin D_2_, which negatively regulates the epithelial response to type 2 cytokines during helminth infections [32].

Although these examples suggest a link between the antimicrobial state of an organ and tuft cell activation, further study is required to determine to which extent tuft cell signaling enables the host to regulate the composition of its eukaryotic and prokaryotic microbiota, and what the consequences are if tuft cell responsiveness is impaired. Increasing resolution of microbial metagenomic analysis and microbiota characterization will be helpful in gaining a deeper understanding of this biology.

### The luminal microbial state regulates tuft cell abundance

Another interesting feature of tuft cell activation is that it can further expand the pool of tuft cells. This effect is most dramatic in the small intestine, where tuft cell-derived effector molecules stimulate IL-13 production by ILC2s, which directly promotes tuft cell (as well as goblet cell) fate in epithelial progenitors [41–43]. The mechanism for this fate specification, however, has yet to be defined. As a consequence of the high cellular turnover in the epithelium of the small intestine, the frequency of tuft cells can increase by more than tenfold within just a few days when luminal parasites are present. In helminth infections, this constitutes an essential feed-forward response circuit that elevates the number of cells that produce effector molecules (including tuft cells); these effector molecules then drive worm expulsion [42, 43]. The intestinal microbial colonization state also determines the abundance of tuft cells in the small intestine, which can reach a stable equilibrium. This equilibrium depends on the cross-talk between tuft cells and ILC2s [25, 41]. Chronic activation of the tuft cell–ILC2 circuit, while generally well tolerated, can significantly impact the small intestinal tissue and can promote an increase in overall small bowel length [25]. Notably, small intestine tuft cell populations can also increase due to perturbation of the bacterial microbiota. This effect was noted in mice treated with streptomycin or polyethylene glycol 3350, in mice with an abrogated secretory cell differentiation, which lack Paneth cells, and in mice deficient in Paneth cell lysozyme (*Lyz1*) [24, 60, 65]. While some of these studies identified increased bacterial succinate production as a driving force, similar to the findings with *Tritrichomonas* protists, the physiological consequences of this response remain unclear. Interestingly, fewer tuft cells were detected in samples from patients affected by inflammatory diseases of the small bowel relative to specimens from unaffected individuals [65, 66].

In comparison to the small intestine, alterations in tuft cell abundance in other tissues are more subtle and the mechanisms that underlie these epithelial remodeling events are less well understood. Mice deficient in TLR2, TLR4, or MyD88—key components in detection of microbial ligands—displayed a reduction in tuft cells in the urethra and trachea, whereas no difference was noted between germ-free and SPF wildtype mice [67]. Moderate expansion of airway tuft cells was found following fungal or mite aeroallergen exposure in mice [68]; a similar increase in abundance was reported for patients with chronic rhinosinusitis with nasal polyposis (CRSwNP) and allergic fungal rhinosinusitis [69, 70]. Further studies are required to determine whether tuft cell sensory function is a necessary upstream event in promoting their own expansion in tissues outside the gut. Notably, tuft cells can also emerge in tissues where they have not previously existed following remodeling associated with metaplasia and inflammation, a phenomenon that was observed in the pancreas and distal lung [71–73]. These findings suggest that diverse changes to the tissue state can promote tuft cell lineage commitment.

It is unclear why epithelial tissues adapt tuft cell abundance to a particular luminal state. One reason could be that it increases both the number of sensory cells and effector molecule sources, thereby lowering the threshold and increasing the amplitude of a response following exposure to the same or related stimulus. De novo differentiation of tuft cells may also provide a mechanism to alter the overall quality of tuft cells, which could be an additional way to finetune tissue responses. Heterogeneity in tuft cells has been reported for the trachea and small intestine, and, in the latter, the composition was shifted following helminth infection [74, 75]. Notably, diversity in small intestinal tuft cells appears to be in part driven by zonated transcriptional programs along the crypt-villus axis [76]. Luminal signals may therefore influence tuft cell heterogeneity at the level of their differentiation from epithelial progenitor cells and/or by directly or indirectly altering gene expression in mature tuft cells. Interestingly, elevated urinary catecholamine levels and increased energy expenditure were found in *Pou2f3*^–/–^ mice which lack all tuft cells and type II taste cells [77]. These findings suggest that changes in certain tuft cell populations might have an impact on organismal energy homeostasis. However, additional work will be necessary to establish such a model.

Overall, these concepts are largely driven by a host-centric view and could be modified to fit a more complex host-parasite relationship. Perhaps, certain parasites deliberately stimulate tuft cells and induce aspects of the abovementioned adaptation to their own benefit, such that they can modify their nutrient accessibility or have a selective advantage over newly invading microbial competitors for the same niche. Interestingly, expulsion of evolutionarily adapted parasites, including some intestinal helminths, is often limited or, in the case of *Tritrichomonas*, even completely absent [41, 78]. Despite their limited expulsion, these organisms stimulate the tuft cell–ILC2 circuit and raise the overall type 2 tissue tone, suggesting a possible benefit of this environment for some parasites. There is, however, clear evidence for enhanced parasite fecundity in the absence of tuft cells or their critical effector molecule, IL-25, indicating an important and dominant host-centric immune function of tuft cells in defending against parasites [42, 79]. Long-term reproductive fitness and success of parasites, however, has not been compared under natural conditions with variable food composition and with many different species competing for similar luminal niches. Under these conditions, stimulation of the tuft cell-ILC2 circuit may suddenly become a beneficial trait for particular parasite species.

## Conclusions and future directions

Studies by many groups over the past few years have established a number of tuft cell agonists, primarily of microbial origin, and a limited repertoire of secreted effector molecules through which tuft cells interact with other cells, including neurons, epithelial cells, and immune cells. While this research identified diverse roles of tuft cells in shaping aversive and adaptive responses to luminal stimuli in different tissues, there is still much more to learn about these cells. In complex organisms, processes in different organs are often cross-regulated and these interactions can induce a state of preparedness prior to exposure to a stimulus that shifts the system away from baseline activity. Hormonal and neuronal pathways are often engaged to promote such anticipatory responses. A classic example is the cephalic phase of digestion when sensory inputs prepare the gastrointestinal tract for the upcoming food processing and nutrient absorption by regulating a significant fraction of the overall secretory activity in organs such as stomach and pancreas before the arrival of food in the gut [80]. Notably, oral bitter taste stimulation can elicit nausea and influence food intake and intestinal motility through effects on a number of physiological pathways, which can be summarized as anticipatory responses in the intestine that confer a prophylactic aversive state [57, 81]. It will be interesting to investigate whether tuft cell activation in one tissue can have physiological consequences in spatially separated tissues. Also, it is unclear if the quality of a stimulus can determine the outcome, i.e., the type of effector molecule that is secreted following ligand detection, and if this is mediated by different types of tuft cells or one cell that is able to discriminate between different agonists. Moreover, the role of immune-related effectors, IL-25 and CysLTs, outside of the small intestine, and the function of acetylcholine produced by tuft cells in the small intestine remain largely unknown. In terms of cellular physiology, the relevance of various interesting morphologic features of tuft cells remains incompletely understood [2, 82]. On the translational side, one can imagine that certain tuft cell signaling pathways may be targetable to increase resilience in particular tissues or to prevent pathologies related to type 2 inflammation.

Over the past years, multiple studies illuminated various aspects of tuft cell biology in diverse tissues and contexts. With the increasing interest in tuft cells stimulating interactions between physiologist, immunologist, and epithelial biologists, we can expect to learn much more about these cells and their impact on organism health in the near future.
